# Poly(4-Hydroxybutyrate): Current State and Perspectives

**DOI:** 10.3389/fbioe.2020.00257

**Published:** 2020-04-03

**Authors:** Camila Utsunomia, Qun Ren, Manfred Zinn

**Affiliations:** ^1^Institute of Life Technologies, University of Applied Sciences and Arts Western Switzerland (HES-SO Valais-Wallis), Sion, Switzerland; ^2^Laboratory for Biointerfaces, Empa, Swiss Federal Laboratories for Materials Science and Technology, St. Gallen, Switzerland

**Keywords:** biopolymer, biopolyester, biobased, 4-hydroxybutyric acid, medicine, implants, tissue engineering, drug delivery

## Abstract

By the end of 1980s, for the first time polyhydroxyalkanoate (PHA) copolymers with incorporated 4-hydroxybutyrate (4HB) units were produced in the bacterium *Cupriavidus necator* (formally *Ralstonia eutropha*) from structurally related carbon sources. After that, production of PHA copolymers composed of 3-hydroxybutyrate (3HB) and 4HB [P(3HB-*co*-4HB)] was demonstrated in diverse wild-type bacteria. The P4HB homopolymer, however, was hardly synthesized because existing bacterial metabolism on 4HB precursors also generate and incorporate 3HB. The resulting material assumes the properties of thermoplastics and elastomers depending on the 4HB fraction in the copolyester. Given the fact that P4HB is biodegradable and yield 4HB, which is a normal compound in the human body and proven to be biocompatible, P4HB has become a prospective material for medical applications, which is the only FDA approved PHA for medical applications since 2007. Different from other materials used in similar applications, high molecular weight P4HB cannot be produced via chemical synthesis. Thus, aiming at the commercial production of this type of PHA, genetic engineering was extensively applied resulting in various production strains, with the ability to convert unrelated carbon sources (e.g., sugars) to 4HB, and capable of producing homopolymeric P4HB. In 2001, Metabolix Inc. filed a patent concerning genetically modified and stable organisms, e.g., *Escherichia coli*, producing P4HB and copolymers from inexpensive carbon sources. The patent is currently hold by Tepha Inc., the only worldwide producer of commercial P4HB. To date, numerous patents on various applications of P4HB in the medical field have been filed. This review will comprehensively cover the historical evolution and the most recent publications on P4HB biosynthesis, material properties, and industrial and medical applications. Finally, perspectives for the research and commercialization of P4HB will be presented.

## Introduction

**FIGURE 1:**
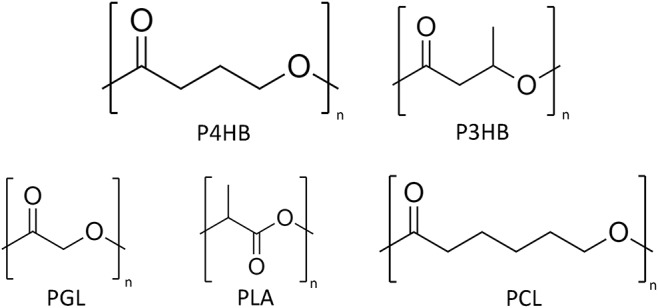
Chemical structures of poly(4-hydroxybutyrate) (P4HB), P3HB, polyglycolide (PGA), polylactide (PLA), and polycaprolactone (PCL).

In 1926, the first polyhydroxalkanoate (PHA), poly(3-hydroxybutyrate) (P3HB) (Figure 1), was discovered by the French scientist Maurice Lemoigne during his work with the bacterium *Bacillus megaterium* (Lemoigne, 1926). In the late 1950s, the first industrial application was considered by W. R. Grace and followed by many other companies (Lenz and Marchessault, 2005). During the past 50 years, much research and development projects have been dedicated to the ever growing class of PHAs. To date, the number of known PHA monomers has increased to more than 140, including unsaturated and aromatic monomers (Kim et al., 2007; Hanik et al., 2019). Nevertheless, there is only limited production of PHA worldwide (Table 1) despite the increase of potential applications (Albuquerque and Malafaia, 2018). The reasons are quite diverse. The high production cost of which approximately 50% are attributed to the precursor substrates, commonly pure sugars and fatty acids, limits the bulk application of PHAs (Koller et al., 2017). These microbial polyesters are estimated to be 3–4 times more expensive than synthetic plastics, such as polypropylene and polyethylene, and exhibit more inconsistent material properties (Kourmentza et al., 2017; Zhang et al., 2018). In addition, the availability of PHAs for process development is limited and, in many cases, the processing methods have to be fine-tuned to a specific polymer type (selection of appropriate additives, temperature profile adapted to optimal crystallization rate, etc.) (Bugnicourt et al., 2014). Last, but not least, stringent patent politics have limited further research and the interest of investors into product development. A window of application where PHAs can outcompete other materials is expected to be the medical field (Zinn et al., 2001; Valappil et al., 2006).

**TABLE 1:** Key players in the commercialization of PHAs today.

**Company**	**Raw material**	**PHA**	**Capacity**	**References**
Kaneka (Japan)	Plant oil	P(3HB-*co*-HHx) (PHBH^TM^)	Estimated production capacity of 12’000 kilotons in 2020	^1^
Tepha Inc. (United States)	Not reported	P4HB (TephaFLEX^®^) and copolymers (TephaELAST^®^)	Not reported	^2^
Danimer Scientific (United States)	Canola oil	mcl-PHA (Nodax^TM^)	Commercial plant (estimated to operate at 10’000 tons per year)	^3^
Tianjin Green Biosciences Co., Ltd., (China)	Not reported	P(3HB-*co*-4HB) (GreenBio)	10’000 tons per year	Greene, 2014;^4^
PHB Industrial S/A (Brazil)	Sugar from sugarcane	P3HB (BioCycle^®^)	50 tons per year	^5^
Bio-on (Italy)	Molasses and by-products of sugar beet production	P3HB (Minerv-PHA^TM^)	Demonstration plant	
Mango Materials (United States)	Raw biogas (methane, carbon dioxide, and hydrogen sulfide)	P3HB	Pilot facility (250 kg per year). Short-term goal: 100 kg per week	

*^1^https://cen.acs.org/business/biobased-chemicals/PHA-biopolymer-whose-time-finally/97/i35; ^2^https://www.tepha.com/platforms/overview/; ^3^https://danimerscien- tific.com/2019/09/16/wincup-to-produce-biodegradable-straws-made-from-danimer-scientifics-nodax-pha-resin/; ^4^http://www.tjgreenbio.com/en/about.aspx?title=Ev- ents&cid=26; ^5^http://www.canaverde.com.br/produtos/.*

New insights in medicine revealed the big potential of resorbable biomaterials in tissue regeneration (Hench and Polak, 2002). A typical representative of this new generation biomaterial is the biopolymer polylactide (PLA) (Figure 1) which has been approved by the United States Food Drug and Administration (FDA) for various medical applications (Tyler et al., 2016). Despite its wide use, PLA has a few shortcomings, e.g., degradation by burst-release and liberating large amounts of lactic acid, which consequently leads to pH acidification of the environment. PHAs, on the other hand, are degraded by enzymatic interaction or by surface erosion via chemical hydrolysis (Martin and Williams, 2003). Out of the PHA family, poly(4-hydroxybutyric acid) (P4HB) (Figure 1) has properties especially suitable for medical applications (Table 2). The extremely high elasticity of P4HB comparable to ultrahigh molecular weight polyethylene is one of its most interesting features (Martin and Williams, 2003). After 10 years of clinical trials, P4HB is unique among all types of PHA produced to date, since it is the only PHA-based material with FDA clearances for clinical usage starting with an approval for monofilament suture applicable in general soft tissue approximation and/or ligation (Williams et al., 2013; Zhang et al., 2018). Since our society, more than ever, has been searching for alternatives to synthetic plastics, the bio-based, biodegradable, and biocompatible P4HB stands out as a material with both high market value as well as large market potential.

**TABLE 2:** Comparison of properties of P4HB with other thermoplastic polyesters [adapted from Martin and Williams (2003)].

**Polymer**	**Tm (°C)**	**Tg (°C)**	**Tensile strength (MPa)**	**Tensile modulus (MPa)**	**Elongation at break (%)**
P4HB	60	−51	50	70	1′000
PGA	225	35	70	6′900	<3
PLLA	175	65	28–50	1′200–2′700	6
PDLLA	^∗^	50–53	29–35	1′900–2′400	6
PCL	57	−62	16	400	80
P3HB	180	1	36	2′500	3

*Depending on the polymer, films were prepared by solvent casting and/or compression molding (Engelberg and Kohn, 1991). ^∗^, Amorphous; PDLLA, copolymer of D- and L-lactic acid; PCL, polycaprolactone.*

## General Aspects of Pha and P4Hb

Polyhydroxyalkanoate is a family of polyesters synthesized by various microorganisms under limitation of a nutrient, besides carbon, essential to cell growth (e.g., nitrogen, phosphorous), and accumulated intracellularly as a stock of energy in the form of PHA granules [also known as carbonosomes (Jendrossek, 2009)]. PHAs are typically divided into three subgroups where the criteria is the number of carbons on the side chain of the repeating monomer unit: short-chain-length PHA (*scl*-PHA) containing monomers up to five carbon atoms (C5), medium-chain-length PHA (*mcl*-PHA) with C6-C14 monomers, and over C15 monomers the polymers are called long-chain-length PHAs (*lcl*-PHA) (Lu et al., 2009). The change of the monomeric unit composition is dictated by the bacterial producer, the type of PHA synthase (PhaC) catalyzing the polymerization, and the substrate fed to the cells, providing PHA with various physical properties (Zinn and Hany, 2005). Once extracted from the cells, PHA can exhibit thermoplastic and elastomeric properties, i.e., *scl*-PHAs have properties resembling polyethylene or polypropylene while the *mcl*-PHAs are comparable to elastomers and rubbers (Sudesh et al., 2000; Koller et al., 2010). Thus, the wide range of physical and material properties of PHAs coming from the variety of monomers makes them suitable as materials for different needs, including value-added medical and pharmaceutical applications (Wu et al., 2009). PHAs are hydrophobic, enantiomerically pure due to the stereo-specificity of PhaC toward (*R*)-3-hydroxyacids (3-HAs), biocompatible, and can be synthesized to molecular weights as high as several millions of g mol^–1^ with low polydispersity (Steinbüchel and Lütke-Eversloh, 2003).

Biodegradation is the decomposition of organic materials/compounds by microorganisms or through biochemical reactions via enzymatic or non-enzymatic hydrolysis both aerobically or anaerobically. In contrast to petroleum-derived plastics, PHAs are considered as intrinsically biodegradable and the chiral center of the PHA monomer in *R* configuration is key for the recognition and hydrolysis of the polymer by enzymes designated PHA depolymerases (PhaZ) (Amstutz et al., 2019). PhaZs are found intracellularly, located on the surface of PHA granules together with other enzymes associated to PHA synthesis, but also extracellularly, including a signal peptide to its protein structure. In addition, lipases, which are thought to present similar hydrolysis mechanism to PhaZs, can also degrade PHAs, particularly the ones without side chains (Ong et al., 2017). Therefore, PHA degradation takes place in environments with high microbial activity (e.g., soil, compost, and marine water) by growing on the polymer surface and secreting enzymes resulting in water-soluble oligomers and HAs which are taken up by the microorganisms. Ultimately, the products of PHA degradation are CO_2_ and H_2_O, and CO_2_ and methane when it occurs aerobically and anaerobically, respectively. Environmental conditions, such as temperature and pH, and PHA properties, likewise monomer type, molecular weight, crystallinity, and surface area, directly influence the degradation rate. In the environment, PHAs are observed to have a relatively fast degradation mostly via enzymatic attack (Jendrossek, 2001). Once in the human body, on the other hand, the degradation of PHAs (e.g., P3HB and P4HB) is mediated by both non-enzymatic and enzymatic hydrolysis into products naturally found as metabolites in animals, favoring their use in medical applications (Deeken and Matthews, 2013; Ong et al., 2017). The hydrolysis of P4HB *in vivo* has been reported to be initiated by the diffusion of water molecules into the polymer matrix cleaving the chains of P4HB (Williams et al., 2016). Polymer surface erosion is driven by enzymes, possibly non-specific lipases and esterases (Brigham and Sinskey, 2012; Williams et al., 2016). PHASIX mesh and P4HB plug, materials made of P4HB for soft tissue repair, are degraded and fully resorbed within 12–18 months (Deeken and Matthews, 2013). The majority of PHAs are composed of 3-HAs, but also 4-, 5-, 6-HAs are polymerized in PHAs. The hydrolytic attack of such PHA ester bonds is less prone to take place in comparison to polymers composed by 2-HAs, such as polyglycolic acid (PGA) and PLA (Figure 1), thus, in general, the hydrolysis rate of PHAs is comparatively slower (Koyama and Doi, 1995).

In 2018, the global production of plastics reached nearly 360 million tonnes (PlasticsEurope, 2019). Around 40% is destined to packaging and half of it is used in food packaging (Albuquerque and Malafaia, 2018). However, the fraction coming from bioplastics (biobased and biodegradable) is almost negligible. In 2016, 0.9 million tons of biodegradable plastics, comprising PHAs, PLA, and starch blends, were produced worldwide and it is expected to increase to 1.3 million tons by 2021 (Albuquerque and Malafaia, 2018). Even though PHAs are suitable materials to traditional polymer processing techniques resulting in daily life products (e.g., packaging, garbage bags, bottles) (Bugnicourt et al., 2014), due to its high cost and interesting material properties, PHAs find relevancy in medical applications. Medical devices, matrices for tissue repair and organ reconstruction, and particles for drug delivery, have been developed using PHAs (Williams et al., 1999; Wu et al., 2009). P4HB, in particular, stands out among both PHAs and polymers conventionally used for medical applications as a material that has the strength of conventional suturing materials but is much more flexible. Undergoing traditional processing techniques (e.g., solvent casting, melting extrusion) and innovative methods, the biocompatible P4HB has been used in the preparation of new and improved biomaterials (e.g., implants, materials for tissue engineering and wound care) filling up the gaps existing when slow *in vivo* degradation is required, for example (Martin and Williams, 2003; Williams et al., 2013). However, limited commercial availability of P4HB might be one of the reasons hindering the exploitation of its huge potential in medical applications.

## P4Hb Production

Commercial P4HB, unlike other resorbable polyesters (e.g., PLA, PGA, PCL), is exclusively synthesized in fermentation process. The chemical synthesis of P4HB has been attempted, but failed to synthesize high molecular weight polymer necessary for most applications (Hori et al., 1995). Furthermore, the use of metal catalysts in the chemical synthesis results in polymers containing toxic metals that are not desired when medical applications are envisioned for such materials.

### Chemical Synthesis of P4HB

One of the chemical synthesis approaches involves the ring-opening polymerization of monomer γ-butyrolactone. Unsuccessful and successful studies were attempted using various catalysts or initiators such as organometallic catalysts (Inoue et al., 1961), zwitterionic titanoxanes (Burlakov et al., 2003), clay catalyst (Miura et al., 1999), metal complex catalyst, and others (Moore et al., 2005). These resulted in oligomers with low degree of polymerization (DP 2 - 3) and polymers with number-average molecular weight (*M*_*n*_) of ∼ 5 × 10^3^ g mol^–1^ (Moore et al., 2005), which do not have the same toughness and flexibility as high molecular weight P4HB produced *in vivo* (over 10^6^ g mol^–1^) (Martin and Williams, 2003). Synthesis of higher molecular weight P4HB with up to 5 × 10^4^ and *M*_*n*_ up to 3 × 10^4^ g mol^–1^ was reported by Oishi et al. (2000) and Hong and Chen (2016), respectively. Oishi et al. (2000) have used Lewis acid catalysts at very high pressure, while Hong and Chen employed as catalyst a homoleptic compound, La[N(SiMe_3_)_2_]_3_, reacted with an alcohol initiator and γ-butyrolactone under ambient pressure. Alternatively, researchers have also developed chemo-enzymatic methods to prepare PHAs. For example, Nobes et al. (1996) disclosed the ring opening polymerization of γ-butyrolactone by mammalian and microbial lipases in *n*-hexane to yield P4HB with *M*_*w*_ up to 932 g mol^–1^. Another synthetic strategy for P4HB is the free radical ring-opening polymerization of 2-methylene dioxolane, resulting in a copolymer containing ring opened and unopened units (Bailey et al., 1982). 4HB has been successfully co-polymerized with 3HB via ring-opening polymerization of γ-butyrolactone and β-butyrolactone using a distannoxane complex as catalyst (Hori et al., 1995). The result, however, was copolymers with low weight-average molecular weight (*M*_*w*_) (less than 1 × 10^5^ g mol^–1^), reaching the lowest values when less than 35 mol% 4HB were polymerized (less than 8 × 10^3^ g mol^–1^). Studies on the ring-opening polymerization for P4HB synthesis were comprehensively reviewed (Moore et al., 2005). Alternatively, as described in the United States Pat. No. 5,563,239, condensation polymerization of esters employing titanium as catalyst under high temperatures was also attempted to synthesize PHAs *in vitro* (Hubbs and Harrison, 1996). More recently, Zhang et al. reported the synthesis of functional P4HB (Zhang et al., 2013). By means of the Passerini multicomponent polymerization of (*E*)-4-oxobut-2-enoic acid with isocyanides followed by hydrogenation, functional P4HB with a *M*_*n*_ up to 8’800 g mol^–1^ were synthesized. By varying the isocyanide compound, two different groups could be incorporated into the side chains of the polymers.

In a polycondensation reaction of 4-hydroxybutyric acids, the formation of lactone is kinetically favored in detriment of the ester bond formation, hampering the synthesis of high molecular weight P4HB (greater than 1 × 10^5^ g mol^–1^) *in vitro* (Niaounakis, 2015). Moreover, γ-butyrolactone has been commonly referred to in the literature as “non-polymerizable.” Such statement was made due to the very small ring strain energy of the cyclic monomer. Due to this, under normal conditions, the extremely stable ester bond in the lactone ring is less prone to break and join onto the polymer chain than the ester in the growing polymer chain is probable to transesterify or undergo ring formation. Nevertheless, even the material resulted from high-pressure ring-opening polymerization reactions of γ-butyrolactone presented a molecular weight not higher than 5 × 10^4^ g mol^–1^ (Moore et al., 2005).

### Biological Synthesis

By varying the bacterium and its PHA polymerase (PhaC) together with the substrate C source, polymers with different monomeric composition and tailor-made mechanical properties are obtained. In addition, PHA producers, either wild-type or recombinant, are capable to synthesize PHAs with molecular weights higher than 10^6^ g mol^–1^. By changing the activity and concentration of active PhaC the molecular weight of PHAs can also be fine-tuned (Hiroe et al., 2012). Many of the catalysts used for the chemical synthesis of polyesters contain toxic metals (e.g., organic tin compounds) and these are completely avoided when using a biological process to produce PHAs.

#### Production of PHA Copolymers Containing 4HB in Wild-Type Strains

Polyhydroxyalkanoates containing 4HB as constituents were first discovered by Prof. Doi’s research group in the late 1980’s when *Cupriavidus necator* (formerly *Ralstonia eutropha*) was cultivated using 4-hydroxybutyric acid and γ-butyrolactone as C sources (Kunioka et al., 1988; Doi et al., 1990). Afterward, biosynthesis of such PHAs was achieved in *Alcaligenes latus*, *Comamonas acidovorans*, *Hydrogenophaga pseudoflava* (Choi et al., 1995), *Burkholderia sacchari* (Cesário et al., 2014; Mendonça et al., 2014), and others (Azira et al., 2011), which are known PHA producers having PhaCs polymerizing preferentially *scl*-PHAs. PHA copolymers containing 3HB and 4HB [P(3HB-*co*-4HB)] could only be synthesized in wild-type strains from structurally related carbon sources, such as 4-hydroxybutyric acid, γ-butyrolactone, 4-chlorobutyric acid (Doi et al., 1988), 1,4-butanediol (BDO), and other ω-alkanediols (Saito et al., 1996). P4HB homopolymer, on the other hand, was hardly produced. Saito et al. (1996) observed the production of P(3HB-*co*-4HB) in *C. necator* from 4HB precursors (e.g., γ-butyrolactone, 1,4-butanediol) as unique carbon sources in nitrogen (N)-free medium. When (NH_4_)_2_SO_4_ and citrate were fed as a substrate mixture together with 4-hydroxybutyric acid, P4HB homopolymer was produced along with a decrease in polymer content from 16 wt% of P(3HB-*co*-13 mol% 4HB) to 2 wt% of P4HB. The metabolism of 4HB-CoA directed to acetyl-CoA rather than 3HB-CoA under growth conditions was a possible reason for the production of P4HB homopolymer. In the same study, *C. acidovorans* JCM10181 (formerly DS-17) was shown to produce P4HB homopolymer with an accumulation of up to 28 wt% from 4-hydroxybutyric acid and 1,4-BDO as sole C sources in N-free medium after 48 h of cultivation. *Alcaligenes latus*, which has a growth-related PHA production (Kourmentza et al., 2017), produced P(3HB-*co*-45 mol% 4HB) from sucrose and γ-butyrolactone. In another publication (Kang et al., 1995), a copolymer with higher 4HB fraction (from 13 up to 83 mol% 4HB) was synthesized in *A. latus* from 4-hydroxybutyric acid and 3-hydroxybutyric acid in a medium containing (NH_4_)_2_SO_4._ Cell growth, however, decreased along with the increase of 4-hydroxybutyric acid concentration in the medium and the higher 4HB content in the polymer. *Comamonas acidovorans* and *A. latus* are not able to grow on 4HB precursors indicating a restricted metabolism of 4HB-CoA to acetyl-CoA. This could explain the production of PHA with a high 4HB fraction in *C. acidovorans*. Although *C. necator* is also not able to grow neither on 4-hydroxybutyric acid nor 1,4-BDO, this strain produces copolymers from these C sources. The metabolism of 4HB in *C. necator* was studied by Valentin et al. (1995). A spontaneous mutant of *C. necator* HF39, denominated SK4040, could be isolated and showed growth on 4-hydroxybutyric acid. After Tn5::mob mutagenesis on SK4040 two classes of secondary mutants were generated, one not able to grow on 4-hydroxybutyric acid (4HB^–^) and a second with poorer growth on 4-hydroxybutyric acid compared to SK4040. A genomic fragment isolated from SK4040 differed from the one of 4HB^–^ mutants. Cloning of such fragment in *C. necator* H16 conferred the wild-type the ability to grow on 4-hydroxybutyric acid. DNA sequence analysis indicated a structural gene encoding a 4-hydroxybutyric acid dehydrogenase and enzymatic assays the existence of an active succinate-semialdehyde dehydrogenase. Thus, 4HB degradation was thought to take place via succinate semialdehyde and succinate followed by degradation through the citric acid cycle. In addition, no evidence for direct conversion of 4HB to 3HB was observed.

A close investigation on the PHA synthase (PhaC_Ca_) of *C. acidovorans* JCM10181 (Sudesh et al., 1998), suggested that the bacterial metabolism rather than a preference of PhaC_Ca_ for 4HB-CoA as substrate is the reason for efficient incorporation of 4HB into PHA. The efficient incorporation of 4HB by *C. acidovorans* JCM10181 was shown to depend on the 4HB precursor supplied (i.e., 4-hydroxybutyric acid, 1,4-BDO, and γ-butyrolactone), among which feeding 4-hydroxybutyric acid resulted in polymer with the highest 4HB fraction (96 mol%) and accumulation up to 25% of the dry cell weight (Lee et al., 2004). Furthermore, reduced culture aeration and higher inoculum concentration to the N-free medium containing the 4HB precursor were found to increase the 4HB fraction in the polymer. An increment in the 4HB fraction (12.3 to 51.8 mol%) in the polymer produced by *C. necator* was obtained when small amounts of propionate along with γ-butyrolactone were added in the medium (Lee et al., 2000). PhaC activity was highly induced by the addition of propionate as well as the concentration of acetyl-CoA. The overflowing acetyl-CoA was proposed to inhibit the ketolysis reaction and thus reducing the lysis of 4HB-CoA to two molecules of acetyl-CoA, consequently leading to an increase of the 4HB fraction available for polymerization. Corroborating with this presumption, the same effect in the 4HB fraction was observed when acetate was supplemented (Kim et al., 2005). The effect of dissolved oxygen tension (DOT) (2 and 20%) and addition of propionic acid (2 g L^–1^) in PHA accumulation and 4HB fraction of a copolymer produced in *C. necator* DSM 545 was investigated in a high cell density fed-batch cultivation (Cavalheiro et al., 2012). Waste glycerol was used as C source for growth (and 3HB generation) and γ-butyrolactone as precursor of 4HB. Higher DOT (20%) favored PHA accumulation and was not significantly increased by the presence of propionic acid. The 4HB fraction, on the other hand, was largely increased by the addition of propionic acid as a 4HB stimulator and by prolonging the cultivation time. However, feeding propionic acid inherently resulted in a terpolymer composed by 3HB, 4HB, and 3-hxdroxyvalerate (3HV). The highest 4HB fraction of 30.6 mol% in a terpolymer containing 6.7 mol% 3HV was achieved with the addition of propionic acid and a DOT of 2%. By optimizing the cultivation conditions, a polymer containing only 4HB was produced in *H. pseudoflava* (Choi et al., 1999). It consisted of a three-step cultivation as follows: 1^st^ stage) biomass formation on LB medium with undesired P3HB accumulation (10 wt%), 2^nd^ stage) degradation of residual P3HB in a C-free medium containing ammonium sulfate, and 3^rd^ stage) synthesis of P4HB homopolymer (19.6 wt%, 0.474 g L^–1^) in a nitrogen-free medium containing γ-butyrolactone for 36 h. It was observed that the activity of 4-hydroxybutyric acid dehydrogenase increased progressively over time in the presence of γ-butyrolactone. In a one-step cultivation at least 120 h were needed for the accumulation of 1.3 wt% of P(3HB-*co*-63 mol% 4HB) from γ-butyrolactone. The activity of 4-hydroxybutyric acid dehydrogenase was 0.089 and 0.557 U mg^−1^ at 96 h and 144 h of one-step cultivation, respectively. The absence of 3HB incorporation into polymer in the three-step cultivation was assigned to the low activity of 4-hydroxybutyric acid dehydrogenase during the short period of PHA accumulation (36 h) leading to low levels of acetyl-CoA.

#### Recombinant Strains for the Synthesis of P4HB Homopolymer and Copolymers From Structurally Related C Sources

P3HB-leaky mutants of *C. necator* JMP222, defective in genes presumably involved in the regulation of P3HB mobilization in the cells (Pries et al., 1991), were found by Steinbüchel et al. (1994) to accumulate low amounts of P4HB homopolymer (up 11.3 wt%) from 4-hydroxybutyric acid. The introduction of a plasmid expressing the PHA biosynthetic genes of *C. necator* into the same mutant increased the cellular P4HB content up to 27.2 wt%. Due to the *phaC* extra copies, recombinant *C. necator* showed approximately five times higher activity than the PHA polymerase (synthase) of the parent wild-type strain, suggesting the limiting-step for the synthesis of 4HB-containing PHA is the PHA synthase rather than the enzyme responsible from the generation of 4HB-CoA from 4HB. Such pattern is different from the understandings on the production of P3HB in *C. necator* in which, instead, the reaction catalyzed by the acetoacetyl-CoA reductase is the rate-determining reaction (Doi et al., 1992). Expression of additional copies of the genes encoding PhaC_Ca_ and β-ketothiolase in *C. acidovorans* JCM10181 resulted in twice-higher P4HB content, from 33 to 63 wt% from 4-hydroxybutyric acid (Sudesh et al., 1999). The genes were cloned in a multi-copy plasmid under the control of the strong hybrid *trc* promoter, PhaC and β-ketothiolase activities were significantly increased. In analogy to findings by Steinbüchel et al. (1994), increased PhaC activity resulted in higher P4HB accumulation. 4-hydroxybutyric acid, a controlled pro-drug, often used as substrate for P4HB production is costly. Knowing the substrate cost represents nearly 50% of the total cost for PHA production (Ahn et al., 2000), 1,4-BDO was used as the starting material to obtain 4HB for P4HB homopolymer production in recombinant *Aeromonas hydrophila* 4AK4, *E. coli* S17-1, or *Pseudomonas putida* KT2442 (Zhang et al., 2009). In this study, 1,4-BDO was converted to 4HB by bacterial cells harboring 1,3-propanediol dehydrogenase gene *dhaT* and aldehyde dehydrogenase gene *aldD* from *P. putida* KT2442. Ten g L^–1^ 4HB was obtained from 20 g L^–1^ of 1,4-BDO after 52 h of cultivation of recombinant *A. hydrophila* in a 6 L fermenter (Zhang et al., 2009). Afterward, this 4HB containing fermentation broth was sterilized and added to minimum or complex media for the production of P4HB and P(3HB-*co*-4HB) in recombinants *E. coli* XL1-Blue and *C. necator* H16, respectively. Here, the necessary two-step fermentation was time consuming and complicated the production process. Moreover, the conversion rate of 1,4-BDO was rather low.

**FIGURE 2:**
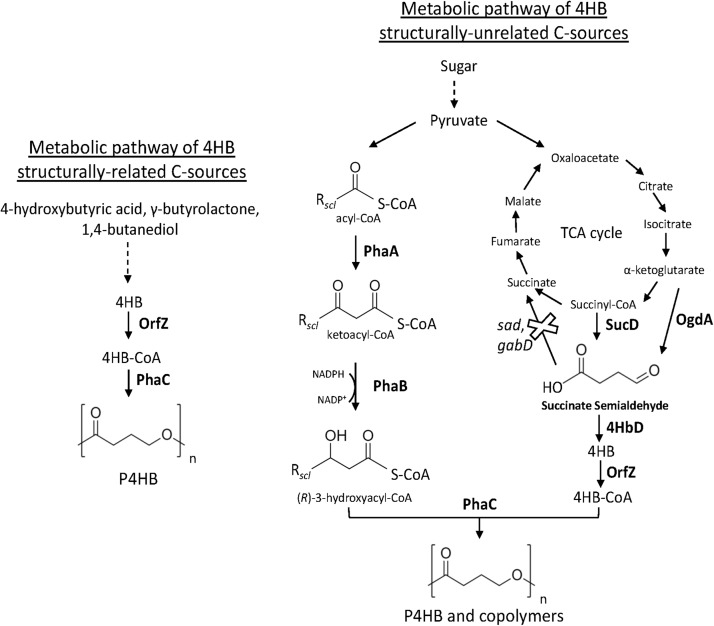
Metabolic pathway heterologously expressed in recombinant bacteria (e.g., *E. coli* and *H. bluephagenesis*) for the synthesis of P4HB and copolymers from related and unrelated C sources. The deletion of endogenous *sad* and *gabD* both encoding succinate semialdehyde dehydrogenase reinforces the carbon flux toward 4HB-CoA. PhaA, 3-ketothiolase; PhaB, acetoacetyl-CoA reductase; OgdA, 2-oxo-glutarate dehydrogenase; SucD, succinate semialdehyde dehydrogenase; 4HbD, 4-hydroxybutyrate dehydrogenase; OrfZ, 4HB-CoA transferase; PhaC: PHA synthase.

Hein et al. (1997) reported the first synthesis of P4HB homopolymer in recombinant *E. coli*. Strains of *E. coli* have been extensively genetically engineered and used in industry for the production of several bioproducts (Katz et al., 2018). Moreover, wild-type *E. coli* is not capable of synthesizing PHA. Thus, the absence of endogenous genes involved in the synthesis of PHA opens possibilities to control PHA monomer composition. The core of the recombinant system of Hein et al. (1997) was the heterologous expression of a gene cluster from the Gram-positive and anaerobic bacterium *Clostridium kluyveri* comprising an open reading frame (ORF) of a putative 4-hydroxybutyril-CoA transferase (OrfZ). This ORF was identified in the studies of Söhling and Gottschalk on the molecular aspects of the succinate degradation pathway of *C. kluyveri* involved in the unusual cofermentation of ethanol and succinic acid in this bacterium in which 4-hydroxybutyril-CoA is an intermediate (Söhling and Gottschalk, 1996). The work consisted on the construction of a genomic library of *C. kluyvery* using *E. coli* as host strain and selection of the clones able to oxidize 4-hydroxybutyrate were selected. A gene region of 6’575 bp was obtained having several ORFs for proteins including succinate semialdehyde dehydrogenase (*sucD*), 4-hydroxybutyric acid dehydrogenase (*4hbD*), succinyl-CoA:CoA transferase (*cat1*), a putative membrane protein (*orfY*), and OrfZ with unknown function by then. In this pathway, CoA is transferred to succinate by CAT1. After, succinyl-CoA is reduced to succinic semialdehyde and CoA in a reaction catalyzed by SucD. Succinic semialdehyde is reduced to 4-hydroxybutyrate by 4HbD followed by CoA activation to 4HB-CoA catalyzed by OrfZ (Valentin and Dennis, 1997). Hein et al. further used *E. coli* XL1-Blue to express *phaC* of *C. necator* and *orfZ* of *C. kluyvery* (Figure 2) in a plasmid called pKSSE5.3 resulting in the synthesis of P4HB homopolymer in a shake flask cultivation. P4HB accumulation of up to 58.5% of its cell dry weight was achieved when 4-hydroxybutyric acid and glucose were fed to the cells. Along with the publication in 1997, a patent was filed by Monsanto Company [publication number: WO199839453 (Hein et al., 1998)]. Some years later in a sequence of studies, P4HB synthesis in *E. coli* using the same plasmid system was further improved via bioprocess optimization. A P4HB content of up to 70 wt% was achieved in single stage batch cultures of *E. coli* JM109 harboring pKSSE5.3 using sodium-4-hydroxybutyrate and xylose as carbon sources (Le Meur et al., 2013). Sodium-4-hydroxybutyrate was obtained from chemical hydrolysis of γ-butyrolactone for production of P4HB in recombinant *E. coli*. The cost for γ-butyrolactone is 250-fold lower than that of 4-hydroxybutyric acid, thus, substantially reducing the production costs. P4HB productivity was also largely improved when exponential feeding of a solution composed by glycerol, acetic acid, and sodium-4-hydroxybutyrate was employed in a fed-batch cultivation employing the same recombinant *E. coli* strain (Le Meur et al., 2014). Amino acid limitation rather than nitrogen limitation was observed to prompt PHA production, thus the addition of a weak organic acid such as acetic acid was strategically supplemented to artificially create an amino acid limitation. As an alternative to acetic acid, the addition of propionic acid along with glycerol and sodium-4-hydroxybutyrate also improved P4HB production in batch cultures resulting in a polymer content of 80 wt% in cell biomass (Kampf et al., 2014). The increase in P4HB accumulation, however, was accompanied by a reduction in residual biomass. Further, the addition of methionine weakened the effect of propionic acid on P4HB synthesis. Therefore, supplementation of propionic acid was suggested to enhance P4HB production by decreasing the intracellular pool of methionine, which results in cell growth inhibition. The consequent increase of acetyl-CoA availability is thought to be the reason for the improved synthesis of P4HB by recombinant *E. coli*.

Halophilic bacteria, which grow in the presence of high concentration of salts, are potential hosts for reducing the costs of PHA production since they can be cultivated continuously under unsterile conditions. In 2017, Chen et al. engineered the marine P3HB producer *Halomonas bluephagenesis* TD01 for the production of P(3HB-*co*-4HB) (Chen et al., 2017). The *orfZ* gene from *C. kluyveri* under the control of constitutive *tac* promoter was integrated into the genome of *H. bluephagenesis* TD01. Recombinant bacterium was cultivated in a medium with glucose and γ-butyrolactone as C sources. Sterilization was not necessary and tap water was used during the whole process. In pilot-scale (1000 L), 61 wt% of P(3HB-*co*-16 mol% 4HB) were accumulated in the cells with a productivity of 1.04 g L^–1^ h^–1^. In a follow up publication (Ye et al., 2018b), *H. bluephagenesis* TD01 was further modified by the deletion of its succinate semialdehyde dehydrogenase gene (*gabD*) resulting in the strain TD40 (Figure 2). Glucose, γ-butyrolactone, and waste corn steep liquor were used as carbon sources for growth and polymer production. The non-sterile fed-batch fermentation was scaled-up to a 5 m^3^ vessel resulting in a cell dry weight of 100 g L^–1^ and accumulation of up to 74 wt% of P(3HB-*co*-13.5 mol% 4HB) after 36 h of cultivation.

#### Production of P4HB Copolymers From Unrelated Carbon Sources in Recombinant Bacteria

The first report on the biosynthesis of 4HB copolymers from unrelated carbon sources was published in Valentin and Dennis (1997). The heterologous expression of genes encoding for SucD, 4HbD, and OrfZ from *C. kluyveri*, along with the genes of the PHA operon of *C. necator* was attempted in *E. coli* (Figure 2). Even though the *lac* promoter was placed upstream the succinate degradation genes, results suggested that the genes were not under the control of this promoter. Using solely glucose as C source, recombinant *E. coli* accumulated up to 46 wt% of P(3HB-*co*-4HB) with no more than 2.8 mol% 4HB. This study indicated that 4HB-CoA is likely to be generated from succinyl-CoA, an intermediary of the tricarboxylic acid cycle (TCA). Improved 4HB incorporation was achieved by further modifying *E. coli* (Li et al., 2010). The strong and constitutive pyruvate decarboxylase promoter (P*_*pdc*_*) of *Zymomonas mobilis* led the transcription of *sucD* and *4hbD* from *C. kluyveri* cloned in a plasmid. Moreover, to hamper the formation of succinate from succinate semialdehyde and reinforce the carbon flux to 4HB-CoA, *E. coli* native succinate semialdehyde dehydrogenase genes *sad* and *gabD* were deleted (Figure 2). Expression of PHA operon genes from *C. necator* and *orfZ* from *C. kluyvery* was driven by the respective native promoters. From glucose and with the addition of α-ketoglutarate or citrate for incrementing 4HB-CoA generation, recombinant *E. coli* accumulated up to 65.5 wt% of P(3HB-*co*-4HB) with a 4HB fraction as high as 20 mol%.

With the motivation to synthesize P(3HB-*co*-4HB) in plants, γ-aminobutyrate (GABA) and glutamate as precursor substrates were evaluated in recombinant *E. coli* (Valentin et al., 2000). The use of plants was considered to be less expensive than bacteria, however, succinyl-CoA is not found at high levels in the plastidial stroma. Glutamate, on the other hand, is an important intermediate of amino acid metabolism in plants. Furthermore, by the decarboxylation of glutamate, catalyzed by glutamate dehydrogenase existing in plants, GABA is generated. GABA is typically degraded via a transaminase reaction resulting in succinic acid semialdehyde that is further oxidized by a succinic acid semialdehyde dehydrogenase to succinate. The genes heterologously expressed by *E. coli* CT101 encoded for glutamate decarboxylase (without calmodulin-binding site) from *Arabidopsis thaliana*, glutamate:succinic semialdehyde transaminase and 4-hydroxybutyrate dehydrogenase from *C. necator*, OrfZ from *C. kluyveri*, and PhaA/PhaB/PhaC of *C. necator*. *Escherichia coli* CT101 does not have NAD^+^-dependent succinic semialdehyde dehydrogenase, reducing the competition with 4-hydroxybutyrate dehydrogenase for succinic semialdehyde. Less than 5% of PHA containing up to 5 mol% 4HB was accumulated in the cells fed with glutamate or GABA. It demonstrates that 4HB-CoA can be generated from glutamate or GABA, however, the metabolic flux in the expressed metabolic pathway was likely to be not enough for the production of higher amounts of P(3HB-*co*-4HB).

Due to the capability to use sunlight as energy source and CO_2_ as the C source, the cyanobacteria *Synechococcus* sp. PCC 7002 was genetically modified to produce copolymers containing 4HB (Zhang et al., 2015), as an alternative production strain to potentially reduce the costs of production. Since succinic semialdehyde is an intermediate in the TCA cycle variant of cyanobacteria, the possibility of 4HB-CoA formation from it was investigated. First, the *phaABEC* operon composed by enzymes for P3HB production from *Chlorogloeopsis fritschii* PCC 9212 was introduced in *Synechococcus* sp. PCC 7002. To avoid the possibility of homologous recombination with the heterologous genes, the operon encoding 2-oxoglutarate decarboxylase and succinic semialdehyde dehydrogenase was deleted as well as the *ccmc* gene (Daley et al., 2012). Lastly, the construct encoding the biosynthetic pathway for 4HB-CoA from succinic semialdehyde comprising 2-oxoglutarate decarboxylase from *Synechococcus* sp. PCC7002, 4-hydroxybutyrate dehydrogenase, and 4-hydroxybutyryl-CoA transferase from *Porphyromonas gingivalis* W83, replaced the chromosomal malate dehydrogenase gene by homologous recombination. From CO_2_, recombinant *Synechococcus* sp. PCC 7002 could accumulate 4.5% P(3HB-*co*-12 mol% 4HB) of its cell dry weight.

Aiming at the synthesis of 4HB copolymers in *H. bluephagenesis* TD01 from glucose, a metabolic pathway was designed for the generation of succinate semialdehyde as direct 4HB-CoA precursor from both 2-oxoglutarate, mediated by 2-oxo-glutarate dehydrogenase (OgdA), and succinyl-CoA, catalyzed by SucD, known intermediates of the TCA cycle (Ye et al., 2018a). The genes *sucD*, *4hbD*, and *orfZ* from *C. kluyveri* and *ogdA* from *Synechococcus* sp. PCC 7002 were then expressed in *H. bluephagenesis* (Figure 2); however, only 0.17 mol% 4HB was incorporated in the polymer produced by this strain. Putative succinate semialdehyde dehydrogenases of *H. bluephagenesis* TD01 were found to deviate the carbon flux by converting succinate semialdehyde to succinate. Among the five putative succinate semialdehyde dehydrogenases identified, double deletion of *gabD2* and *gabD3* increased 24-fold the 4HB fraction, while the deletion of the respective five genes did not further increase the incorporation of 4HB. In a batch cultivation, the double knockout mutant accumulated up to 60.5 wt% of P(3HB-*co*-4HB) with a maximum of 25 mol% 4HB from glucose.

#### Production of P4HB Homopolymer From Unrelated Carbon Sources in Recombinant Bacteria

In 2004, Metabolix Inc., a company founded by Oliver P. Peoples and Antony Sinskey from the Massachusetts Institute of Technology (MIT), patented the invention of synthesis of PHA containing 4HB, including P4HB homopolymer, by transgenic organisms (Huisman et al., 2002). This patent describes the generation of transgenic microorganisms, e.g., *E. coli*, and plants by the integration of genes needed for the production of 4HB containing PHAs on the chromosome. The expression of genes of interest is sufficient and stable once the transcriptional and translational signals preceding the genes were optimized. It is also claimed that the use of inexpensive carbon sources (e.g., glucose, sucrose, xylose, and lactose) is enhanced by increasing enzyme activities in the γ-hydroxybutyrate shunt. At the same time, reducing the activity of enzymes that drain the intermediates of 4-hydroxybutyric acid from this shunt is reduced. Moreover, methods to redirect the cellular metabolite succinic acid to 4-hydroxybutyric acid were also developed. Because engineered *E. coli* K12 has been characterized for production of medical compounds and it is highly efficient, to the best of our knowledge, 50 g L^–1^ of polymer is produced in less than 48 h (Martin and Williams, 2003). This strain is used for the production of P4HB, which is commercialized with the tradename TephaFLEX^®^. In 2016, the producer of TephaFLEX^®^, Tepha Inc. (Cambrigde, MA, United States), completed a buyout of its royalty obligation to Metabolix Inc., making Tepha Inc. the current patent-holder of US6689589.^1^

Zhou et al. (2012) reported hyperproduction of P4HB homopolymer from unrelated C source, i.e., glucose, in a recombinant strain. *Escherichia coli* JM109 with knocked out native genes (*sad*, *gabD*) encoding succinate semialdehyde dehydrogenase, heterologously expressed in plasmids the genes encoding for succinate degradation enzymes (*sucD*, *4hbD*, and *orfZ*) of *C. kluyveri* and PhaC of *C. necator* (Figure 2). In addition, individual expression of four phasins of *C. necator* (*phaP1*, *phaP2*, *phaP3*, and *phaP4*) and their effect on the synthesis of P4HB was investigated. Phasins are proteins located on the surface of PHA granules and participate in the regulation of surface/volume ratio of the granules but also interacting with the PHA synthase. Among the four phasins, PhaP1, followed by PhaP3, led to the highest PHA accumulation. Up to 68.2 wt% of P4HB was accumulated from glucose in recombinant *E. coli* expressing *phaP1* (Zhou et al., 2012). Indeed, PhaP1 is the major phasin of *C. necator* (Pötter et al., 2005). Single knockout of *phaP1* significantly decreased P3HB accumulation while the single knockout mutants of *phaP2*, *phaP3*, and *phaP4* had similar accumulation to the wild-type *C. necator* H16 (Pötter et al., 2005). To summarize, Table 3, exhibits patents on the biosynthesis of P4HB and copolymers.

**TABLE 3:** Patented methods for the production of PHAs containing 4HB.

**Publication number**	**Content**	**References**
WO2018233703	Gene cassette for fine control of composition ratio of 4-hydroxybutanoic acid in copolymer and application thereof	Li et al., 2018
WO2014058655	Polyhydroxyalkanoate copolymer compositions (3HB-*co*-4HB) and methods for making the same	Ramseier et al., 2014
US6689589	Biological systems for manufacture of polyhydroxyalkanoate polymers containing 4-hydroxyacids	Huisman et al., 2002
US6117658	Methods of making polyhydroxyalkanoates comprising 4-hydroxybutyrate monomers units	Dennis and Valentin, 2000
WO1998039453	Methods for the biosynthesis of polyesters	Hein et al., 1998
WO1997007153	Methods for controlling microbial polyester structure by the addition of polyethylene glycol (PEG)	Gross et al., 1997

#### Monomer Composition and Microstructure Control of P(3HB-*co*-4HB)

Clustered regularly interspaced short palindromic repeats interference (CRISPRi) was evaluated as a strategy to adjust the monomer composition of P(3HB-*co*-4HB) using solely glucose as C source (Lv et al., 2015). The CRISPR/Cas system comprises a Cas9 endonuclease and a single guide RNA (*sg*RNA) complementary to a genomic target sequence. This system generates a double strand break at a specific site of the genome, a feature that has been extensively used for gene editing in bacteria and eukaryotic cells. In CRISPRi, Cas9 has its endonuclease function inactivated (dCas9), but it still binds to a target genome loci directed by the *sg*RNA. Gene transcription can be blocked by the binding of dCas9-*sg*RNA complex to the upstream region of the gene (Cho et al., 2018). *Escherichia coli* was engineered to express the genes from *C. necator* PHA operon and *orfZ*, *sucD*, and *4hbD* from *C. kluyreri*. The *E. coli* genes encoding succinyl-CoA synthetase (*sucC* and *sucD*), succinate dehydrogenase (*sdhA* and *sdhB*), and succinyl-CoA synthetase (*sad*) were individually, in pairs, or simultaneously downregulated using the CRISPRi system in order to strengthen the carbon flux in *E. coli* toward 4HB-CoA synthesis. 4HB fraction in the polymer increased, as more genes were downregulated simultaneously. Reduced expression of all five genes resulted in the highest 4HB fraction of more than 18 mol%.

*Pseudomonas putida* KTOY08ΔGC, a weakened β-oxidation mutant of *P. putida* KT2242 having the genes encoding PHA synthase (*phaC*) and 3-hydroxyacyl-CoA-acyl carrier protein transferase (*phaG*) knocked out, was used as host strain aiming at the synthesis of PHA block copolymers of 3HB and 4HB (Hu et al., 2011). Due to microphase separation of PHA blocks with distinguished physical properties, structurally controlled PHAs as such can present novel properties compared to random and blend copolymers. The synthesis of which, however, is not trivial. *Pseudomonas putida* KTOY08ΔGC was modified to express *phaC* of *C. necator* and *C. kluyveri orfZ*. Because of its reduced β-oxidation metabolism, this strain was able to use sodium-butyrate and γ-butyrolactone for PHA production more efficiently. Sequential feeding of both substrates was the strategy used for making PHA block copolymers. In a cultivation in LB medium, γ-butyrolactone was added at 12 and 36 h of cultivation leading to the formation of P4HB. Sodium-butyrate was fed to the cells at 60 h and the fermentation proceeded for another 48 h with the formation of the second block composed of pure 3HB. The block structure of the PHA with 80 mol% 4HB was assessed by NMR analyses and the thermal and mechanical properties of the polymer were also examined.

## Downstream Processing of P4Hb

The downstream processing (DSP) of PHAs, which comprises the recovery of PHA-containing biomass from the cultivation broth until the final polymer at a certain level of purity depending on the material applications, is one of the most expensive steps of PHA production. However, little progress has been observed compared to the innovations on metabolic engineering and bioprocess. In order to separate PHA from cell components (e.g., cell envelope, nucleic acids, and peptides) and from PHA granule-associated proteins, appropriate extraction processes must be applied. Different processes have been investigated and established in the past. The conventional method involves an organic solvent to extract the water insoluble polymer from dry biomass, which is then precipitated in a non-solvent resulting in the removal of also oligomers and monomers. The treatment of the cells with an aqueous mixture of chemical agents (e.g., NaOH and KOH) and/or enzymes to lyse bacteria obtaining PHA as latex is also commonly used. Jacquel et al. (2008) and others Furrer et al. (2007) have published an excellent summary of the different extraction methods for PHAs. The often-used solvents to recover PHAs from freeze-dried biomass include chloroform, methylene chloride, tetrahydrofuran methyl anhydride, tetrahydrofuran ethyl cyanide, and others were tested. Although the solvent extraction method is able to give a high level of purity, it is difficult to separate solubilized PHA from non-PHA cell materials when only filtration is applied. Even though pressure can be applied, the separation often takes a long time and filters are often blocked. Extraction using organic solvents results in PHA with higher purity than when aqueous treatment is employed. A key factor to the application of PHAs in medicine is the appropriate removal of contaminating pyrogenic compounds, which can cause inflammation and fever in humans. The endotoxins (lipopolysaccharides) are the components of the outer cell membrane of Gram-negative production strains (Petsch and Anspach, 2000). The United States Pharmacopeia (USP) sets as a threshold an upper limit of 20 endotoxin units (EU) per medical device, and 2.15 EU per device that contacts the cerebrospinal fluid (F.D.A., 1997). Lee et al. (1999) compared chloroform extraction and NaOH digestion method for recovering P3HB from cells of gram-negative bacteria (*C. necator*, *A. latus*, and recombinant *E. coli*) concerning the removal of endotoxins. P3HB obtained from the chloroform extraction method by the addition of 50 volumes of chloroform and incubation at 30°C from 48 h resulted in less than 10 EU per g of P3HB (Lee et al., 1999). By using the NaOH digestion method at 30°C, either 0.2 N NaOH for 5 h or a NaOH concentration higher than 2 N, polymer with less than 1 EU per g was recovered (Lee et al., 1999). A temperature-controlled precipitation method (Furrer et al., 2007) as well as the addition of active charcoal at the beginning of polymer extraction with ethyl acetate (Wampfler et al., 2010) have also been reported to lead the recovery of *mcl*-PHAs synthesized in *P. putida* species with less than 2 EU per g of polymer.

Most of the methods depicted in Table 4, have been mainly developed to isolate and purify *scl*-PHAs such as P3HB and P(3HB-*co*-3HV). Thus, the same methods can be extrapolated for the purification of P4HB as well. All these methods have both advantages and disadvantages. To the best of our knowledge, methods for P4HB extraction have been mainly solvent-based from freeze-dried biomass. Due to the disadvantages of the solvent extraction method, development of cost-efficient, easy-to-handle, scalable, and eco-efficient process for P4HB purification is indispensable for enlarging the use of this polymer in diverse applications.

**TABLE 4:** Patents for downstream processing of PHA.

**Publication number**	**Brief description**	**References**
US20190127727A1	Application of high-voltage pulsed electric field to the waste sludge during the extraction step to destroy the microorganisms and release the PHAs	Tsai et al., 2019
WO2005052175A2	Process for recovering PHAs from cellular biomass using non-halogenated compounds	Mantelatto et al., 2005
WO2006031492A1	Single solvent polymer extraction methods	Anderson et al., 2006
US2005287654A1	Process for the solvent-based extraction of polyhydroxyalkanoates from biomass	Narasimhan et al., 2005
US2001006802A1	Methods for separation and purifying polyhydroxyalkanoates	Horowitz and Brennan, 2001
US6087471	High temperature PHA extraction using PHA-poor solvents	Kurdikar et al., 2000
US5894062	Process for the recovery of polyhydroxyalkanoic acid	Liddell, 1999
WO9846782A1	Methods of PHA extraction and recovery using non-halogenated solvents	Kurdikar et al., 1998
US5821299	Solvent extraction of polyhydroxy-alkanoates from biomass facilitated by the use of marginal nonsolvent	Noda, 1998b
US5849854	Process for recovering polyhydroxyalkanotes using air classification	Noda, 1998a
WO9707230A1	Solvent extraction of polyhydroxy-alkanoates from biomass	Noda and Schechtman, 1997
US4101533	Cyclic carbonic acid esters as solvents for poly-(β)-hydroxybutyric acid	Lafferty and Heinzle, 1978
US3044942	Process for preparing poly-β-hydroxybutyric acid	Noel, 1962

## Material Properties of P4Hb

P4HB is a strong thermoplastic (i.e., it is moldable when heated and hard at room temperature) and significantly more flexible than synthetic resorbable polymers such as PGA and poly-L-lactide (PLLA). Its tensile strength is comparable to ultra-high molecular weight polyethylene and an elongation at break of around 1000% (Martin and Williams, 2003). After stretching P4HB, the mechanical strength increases but the material remains flexible. The same does not occur with PGA or PLLA, which mechanical strength rises along with polymer brittleness (Williams et al., 2016). As for other polymers, the processing history of the material influences the mechanical properties. The mechanical strength of P4HB increases to a large extend with the orientation of the fibers produced though conventional melt spinning processes and which can be further processed using textile methods such as braiding, knitting and weaving. Compression molded P4HB films, on the other hand, presents high elongation at break similar to polycaprolactone (PCL) (Figure 1), but lower tensile strength than the aligned polymer chains in fibers (Williams et al., 2013). Another interesting feature of P4HB is the possibility to modify the polymer properties *in vivo* by the incorporation of another monomer such as 3HB by the bacterial PHA synthase. For example, copolymers of 4HB and 3HB with a 4HB fraction of 20–35% are elastomeric which can extend and return with force (Martin and Williams, 2003). P4HB has a melting temperature (*T*_*m*_) of 60°C and a glass transition temperature (*T*_*g*_) of −51°C (Table 2). When melted up to 200°C P4HB is reasonably stable presenting only a moderate molecular mass loss (Martin and Williams, 2003). Kunioka and Doi (1990) investigated the thermal degradation in the range of 100 to 200°C for P(3HB-*co*-HV) and P(3HB-*co*-4HB) with different monomer compositions of up to 71 mol% HV and 82 mol% 4HB, respectively. The polymers presented time-dependent changes in molecular weight following the kinetic model of random chain scission at ester groups and were considered thermally stable at temperatures up to 160°C. The rates of random scission were independent on the polymer monomer composition but strongly dependent on temperature. Thus, P4HB has a large thermo processing window and methods applicable to thermoplastics can be readily used to process P4HB according to the desired shape, microstructure, and properties. Mitomo et al. have determined the amorphous density of P4HB as 1.213 in comparison to 1.179 for P3HB. With this data, the heat of fusion, the necessary heat to melt the crystallites, could be calculated as 76 J g^–1^ for P4HB and 125 J g^–1^ for P3HB (Mitomo et al., 2001). The heat of fusion of P4HB is similar to that of PCL (78.1 J g^–1^). An enzymatic degradation assay of P(3HB-*co*-4HB) with various 4HB contents (0–90%) was also performed using PHA depolymerase from *Ralstonia pickettii* T1. The polymer with 4HB fraction of 15 or 24% had the fastest degradation rate while the slowest degradation rate occurred using the polymer with 90% 4HB. The degradation rate by the PHA depolymerase decreased with the decrease of polymer crystallinity and 3HB fraction.

### Molecular Mass Influences the Material Properties

**FIGURE 3:**
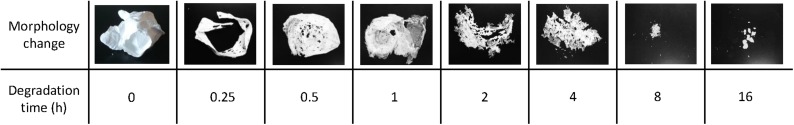
P4HB morphological change along a polymer degradation assay using a solution of sulfuric acid in methanol as catalyst for 16 h. Adapted from Boesel et al. (2014).

Since there are not many producers of P4HB, consequently it is difficult to obtain material samples for property analysis. Based on research studies, P4HB synthesized in recombinant *E. coli* JM109 expressing pKSSE5.3 can result in ultra-high molecular mass (*M*_*w*_ ∼ 2.0–2.5 × 10^6^ g mol^–1^) (Boesel et al., 2014). Polymer processing via solvent- or melt-processing can be stunted by such high molecular weight. Further degradation by chemical method using a solution of sulfuric acid in methanol as catalyst added at every degradation time point for 16 h led to a change in properties and morphology (Figure 3; Boesel et al., 2014). P4HB degradation resulted in polymers with a *M*_*w*_ ranging from 2.5 × 10^6^ to 9.4 × 10^3^ g mol^–1^ (Table 5). Interestingly, a decrease in molecular weight resulted in an increase in polymer crystallinity without an effect on neither melt nor glass transition temperatures. Moreover, both tensile strength and modulus decreased along with the decrease in molecular weight. Therefore, by carefully controlling P4HB molecular weight the mechanical properties can be modified according to the envisioned applications.

**TABLE 5:** Molecular weights of P4HB after degradation by acidic methanolysis. Adapted from Boesel et al. (2014).

	**Degradation time (h)**							
**Molecular weight**	**0**	**0.25**	**0.5**	**1**	**2**	**4**	**8**	**16**
*M*_*n*_ (g mol^–1^)	872’134	292’365	89’267	49’481	30’245	16’791	9’940	6’006
*M*_*w*_ (g mol^–1^)	2’500’000	917’588	167’885	92’945	54’780	29’791	17’446	9’441

## Biocompatibility and Biodegradability of P4Hb

P4HB is known not only as cytocompatible but also very well tolerated *in vivo* (Martin and Williams, 2003). Indeed, the hydrolysis of P4HB yields 4HB which is naturally found in the human body (i.e., brain, heart, lung, liver, kidney, and muscles) (Nelson et al., 1981). This metabolite has a half-life time of about 35 min, and is degraded in the body via the Krebs cycle and subsequently converted to carbon dioxide and water (Sendelbeck and Girdis, 1985). Furthermore, since 4HB has a high p*K*_*a*_ [4.72 at 25°C (Lide, 2005)] and is prone to lactonization, it is less acidic than the α-hydroxy acids such as glycolic [p*K*_*a*_ 3.83 at 25°C (Serjeant and Dempsey, 1979)] and lactic acid [p*K*_*a*_ 3.86 at 20°C (O’Neil, 2013)] that are released from PGA and PLLA implants, respectively. The pharmacology of 4HB is well understood (Martin and Williams, 2003). Sodium 4HB, designated in the medical/pharmaceutical fields as sodium γ-hydroxybutyrate (GHB) or sodium oxybate, is a prescription medicine marketed as Xyrem^®^ for the treatment of cataplexy attacks in patients with narcolepsy. The administration of 4.5 g of Xyrem^®^/night divided into two doses of 2.25 g with interval of 2.5–4 h, with a medication increase of up to 9 g of Xyrem^®^/night has FDA clearance (Carter et al., 2009). However, 4HB itself is classified as a Schedule I substance by the FDA with no accepted medical use and high potential for abuse.^2^ Xyrem^®^, on the other hand, is a Schedule III drug with moderate to low potential for physical or psychological dependence.^2^ In the last decades, the illicit use of 4HB as a “club drug” causing euphoria and relaxation is of major concern. On top of it, 4HB color- and odorless appearance facilitate the use for sedation (Drasbek et al., 2006). Amnestic and variable sedative effects are observed under low doses of 10–20 mg 4HB kg^−1^ body weight while higher doses of 30–40 mg kg^−1^ or higher is reported to cause bradycardia, respiratory depression, and coma (van Noorden et al., 2016; Le and Richards, 2019). The effects become even more acute when 4HB is taken with other intoxicants, such as alcohol (Le and Richards, 2019). Due to the relatively slow rate of P4HB degradation to 4HB and short half-life time of 4HB in the body, small P4HB implants and devices are not expected to induce undesired pharmacological effects (Martin and Williams, 2003; Drasbek et al., 2006). Nevertheless, the biosynthesis of P4HB from 4HB and structurally related C sources (e.g., γ-butyrolactone, 1,4-BDO) is not feasible in large scale and the research on it becomes limited for safety reasons.

It has been reported that P4HB degrades more slowly than PGA, but faster than PLLA, and PCL in a non-specified subcutaneous environment (Martin and Williams, 2003). In one implantation study, for patch augmentation of the pulmonary artery in a juvenile sheep model, the loss of mass from the implanted P4HB patch varied with surface porosity (Stock et al., 2000). Increasing porosity resulted in the increase of degradation rate, proposing that degradation of P4HB *in vivo* depends to some extent on the contact area. Furthermore, it has been observed that implants of P4HB are likely to undergo gradual changes in mechanical properties different from other synthetic absorbable polymers, for example PGA, in which the mechanical properties can be remarkably changed at sudden (Bostman, 1991). This also impacts on the slow and gradual release of 4HB in the blood, which avoids high acidification of the environment and accumulation of it in the body. In large PGA implants, on the other hand, a rapid loss of implant strength leading to the accumulation of highly acidic degradation products is likely to result in severe foreign body reactions (Bostman, 1991). Thus, P4HB’s gradual biodegradability, which results in natural metabolites well tolerated in the body, gives to this material an *in vivo* stability status that is a major breakthrough in the fabrication of medical implants and scaffolds.

## Applications of P4Hb

P4HB is a thermoplastic material that can be processed into various shapes and forms including fibers, films, tubes, foams, textiles, microspheres, and molded constructs using standard processing techniques [see review (Martin and Williams, 2003)]. Table 6 displays examples of patents on P4HB in diverse medical applications.

**TABLE 6:** Examples of patents on the applications of P4HB and copolymers thereof.

**Publication number**	**Content**	**References**
WO2015006737A1	Absorbable implants for plastic surgery	Felix et al., 2015
WO2012142100A1	Biodegradable coextruded multilayer films	Krishnaswamy, 2012
WO2008070428A2	Medical devices containing oriented films of poly-4-hydroxybutyrate and copolymers	Rizk et al., 2008
WO2007092418A3	Polymeric, degradable drug-eluting stents and coatings comprising copolymers or homopolymers of 4-hydroxybutyrate	Behrend et al., 2007
WO2006081517A2	Embolization of poly-4-hydroxybutyrate particles	Martin et al., 2006
WO2004101002A2	P(4HB) fiber useful in devices such as medical textile, tube, general surgical mesh, hernia mesh, pericardial patch, anti-adhesion patch	Martin et al., 2004
WO2001015671A2	Drug delivery devices or bandages	Williams, 2001
WO2001019422A1	Polyhydroxyalkanoate compositions for soft tissue repair, augmentation, and viscosupplementation	Williams and Martin, 2001

### P4HB for Medical Implant Materials and of Regenerative Medicine

A persisting challenge after the development of the first synthetic absorbable material for medical applications in 1970 was their extremely fast degradation in the body, thus a relatively short strength retention *in vivo*. Such polymers were successfully used in wound healing, but not as scaffolds for long-term reinforcement. After the FDA clearance of an absorbable monofilament suture in 2007, P4HB became the first long-term resorbable implant to enter the market in many years (Williams et al., 2016). Today, Tepha Inc. possesses 510(k) FDA clearance for medical devices made for their produced P4HB and products are currently available in the United States and Europe. The portfolio of products made from TephaFLEX^®^ polymer is composed by absorbable monofilaments (suture, mesh, and fiber), absorbable surgical film, and composite mesh are under development by Tepha Inc. absorbable multifilament fiber, absorbable knitted multifilament mesh, absorbable nonwoven textiles, cardiovascular absorbable stents and stent coatings from TephaFLEX^®^ but also from their elastomeric absorbable TephELAST^®^ polymer.^3^ In collaboration with Tepha Inc., B. Braun Melsungen AG (Melsungen, Germany) fabricates Monomax^®^, a monofilament for synthetic suture.^3^ The use of P4HB as medical implants has been extensively reviewed by Martin and Williams (2003) and Williams et al. (2013).

Tissue engineering aims at repairing damaged or replacing diseased tissues and organs. Tissues can be classified as hard tissues (bone and cartilage) and soft tissues (e.g., vascular and skin grafts). The biomaterial used should have mechanical properties for supporting the organ during tissue regeneration and own an appropriate surface topography for cell adhesion and proliferation (Rodriguez-Contreras, 2019). P4HB scaffolds are used for soft tissue support, concomitantly allowing robust tissue ingrowth. The material is resorbed in a predictable and steady manner (Williams et al., 2016). A wholly owned company of Tepha Inc., Galatea Surgical Inc. (Lexington, MA, United States) fabricates absorbable surgical scaffolds for soft tissue support and repair.^4^ Collaborators of Tepha Inc., C. R. Bard Inc. (New Jersey, United States) commercializes Phasix^TM^ Mesh, a resorbable implant for soft tissue reconstruction and the absorbable scaffold BioFiber^®^ is commercialized by the Wright Medical Group Inc. (Memphis, Tennessee, United States)(see text footnote 3). In addition, the creation of a scaffold for a trileaflet heart valve made from P4HB have also been reported. The scaffold functioned appropriately for 120 days in lambs, indicating the suitability of P4HB for tissue engineering of heart valves (Sodian et al., 2000). Moreover, researchers of the University of Zürich and collaborators have been exploring the potential of P4HB-based biomaterials and now have the approval for clinical studies. Stented trileaflet heart valves fabricated from PGA-P4HB composite matrices aiming at prenatal cardiac interventions (Weber et al., 2012), PGA-P4HB-based valves incorporated into metallic stents for cardiac and venous valve repair (Ksia̧żek et al., 2016), and an anisotropic and porous 3D PGA microfibrillar scaffold coated with P4HB to promote anisotropic cellular organization for surgery and tissue engineering (Hosseini et al., 2018), are among the investigated biomaterials with promising applications.

### P4HB as Drug Delivery Carrier and Microspheres

Biodegradable polymeric nanoparticles (NP), which are polymeric colloidal particles with their size ranging in one dimension from 1 to 100 nm, have been studied for application in drug delivery (Som et al., 2019). The potential advantages are the controlled and sustained release of an encapsulated drug driven by a biodegradable and biocompatible material to its specific site. The use of such system can avoid side effects as observed in the treatment of tumors associated with multiple dosing of the drug (Duncan, 2006). Last but not least, nanoparticles cross the cellular membranes of body much easier than microparticles. Since P4HB is an approved material for diverse medical applications in the United States and European countries, preparation and validation of NP for drug delivery from P4HB is of high interest. Shah et al. (2014) reported the preparation of NPs from amphiphilic block copolymers of P4HB and monomethoxy polyethylene glycol (mPEG) (P4HB-mPEG) encapsulating the anticancer drug cisplatin. PEG is extensively used as hydrophilic non-toxic segment approved by the FDA for human use. NPs with mPEG incorporated on their surface have shown resistance against opsonization and phagocytosis, besides its prolonged residence time in blood. The surface modification also avoids the recognition of the NPs by macrophages, thus enables the sustained release of the encapsulated drug. Amphiphilic block copolymers possess the physicochemical characteristics of self-assembly and stability in aqueous solution. Hydrophobic drug molecules solubilize within the hydrophobic core while the shell protects the integrity of the micelle. For the fabrication of nanoparticles, low molecular weight block copolymers (*M*_*w*_ 5 × 10^3^–9 × 10^3^ g mol^–1^) were used (Shah et al., 2010). Cells of mouse hippocampal HT22 were treated with the fabricated NPs with and without encapsulated cisplatin, an anticancer agent. Suppression effect on the cell growth of HT22 was observed by flow cytometry and confocal microscopy when cells were treated with cisplatin loaded nanocarriers. Moreover, cell apoptosis observed using NPs with cisplatin encapsulated was comparable with the situation when the free drug was used to treat the cells. Similarly, copolymers of 4HB with 3HB or 3HV were also used for preparing nanocarriers (Shah et al., 2010, 2012). Tepha Inc. patented in 2005 (publication number: United States 20050025809A1) controlled release systems using nanospheres, microparticles, and microcapsules of P4HB and copolymers for sustained drug delivery. On Tepha’s website, matrices for drug delivery from P4HB is described as under development. Absorbable microspheres for tissue bulking procedures, endovascular embolization, and cosmetic surgery are also reported as potential bioproducts of P4HB (see text footnote 2). Recently, Zhang et al. (2018) and Rodriguez-Contreras (2019) published comprehensive reviews on medical applications of PHAs.

## Future Challenges of P4Hb in the Market

Among other, two aspects are highlighted to tackle the expansion of P4HB commercial applications. One is lowering the production costs of P4HB and the second is to identify high value added applications for P4HB in complement to the medical use of this material. Similar to the rapid development of PLA promoted by NatureWorks LLC (Minnesota, United States) as a bulk bioplastic, which is today one of the most representative bio-based plastics, better availability of P4HB will speed up its development into new materials with unique properties. To lower P4HB production costs, isolation and selection of robust P4HB native producers and/or strain genetic engineering with a rational design for the efficient synthesis of P4HB having as a basis the large scientific progress done in this direction as reviewed in this paper. A major challenge is the high and stable synthesis of P4HB from inexpensive/structurally unrelated carbon sources, which is only achievable in recombinant strains. In addition, bioprocess engineering to implement an optimized process on inexpensive medium and substrates is essential to increase the productivity and reduce the costs. Finally, environmentally friendly, simple, efficient, inexpensive, and scalable methods for extraction of P4HB from cells resulting in polymers with a range of purity, including the ones considered endotoxin-free, are yet to be reported and would largely contribute to improve the overall process of P4HB production (Resch et al., 1998; Gao et al., 2013). Synthesis of P4HB with tailored molecular weights according to requirements for different applications would expand even more the uses of this material. P4HB with ultra-high molecular weights of over several millions has been biosynthesized (Boesel et al., 2014). To date, it is not clear yet what determines the molecular weight and distribution of PHA produced in native and recombinant bacteria. The concentration and catalytical activity of PhaC, the occurrence of chain transfer reactions, and if PHA biosynthesis takes place or not in parallel to degradation, are known to play a role in determining the PHA molecular weight (Tsuge, 2016). Ultra-high molecular weight PHA can be turned into high strength fibers. On the other hand, low molecular weight P4HB could be employed for example for drug delivery and as plasticizer. However, these and many other properties are underexplored because of the limited availability of this material due to patent protection and high cost in comparison to the biopolymers produced globally. An increase in production volume would trigger a cheaper correct to price and contribute to a significant increase of variety also with cost-effective applications of P4HB: A classical chicken and egg situation. In order to establish both, a sustainable demand and supply of P4HB, the increase of the relevancy of P4HB, meaning an increase of the visibility along with a gain of knowledge triggered by more intensified research. Along with the scale-up of production, a suitable distribution network with customer service explaining the particular processing of P4HB has to be implemented. In counterpoint, even though P4HB has the FDA approval for medical applications, the increase of P4HB accessibility could give rise to its abusive uses.

## Conclusion

More than 30 years after the discovery of a copolymer containing 4HB and despite the wide range of potential applications in industry, to date, the medical applications of P4HB are still the most economically practical area. P4HB has been studied mostly for bio-implant applications showing good biocompatibility, biodegradability, appropriate stability, and good mechanical properties especially with respect to strength and ductility. After 10 years of clinical trial P4HB received its first FDA clearance in 2007. Currently, P4HB is the only PHA that can be commercially used for medical applications and the reasons for it includes the time and investment required for receiving the FDA medical approval. Reduction of production costs, expansion of P4HB utilization in more routine applications (like it is the case for PLA) which could come across approval constraints (due to the psychoactive effects of the degradation product 4HB), and the development of new biomaterials for various smart medical and therapeutical applications, are on the future path of the auspicious biopolyester P4HB.

## Author Contributions

CU and QR wrote the manuscript. QR and MZ contributed with critical feedback and revisions. All authors contributed to the manuscript revision, read and approved the submitted version.

## Conflict of Interest

The authors declare that the research was conducted in the absence of any commercial or financial relationships that could be construed as a potential conflict of interest.

## Footnotes

1 https://www.tepha.com/news-events/press-releases/post/august-31st-2016-tepha-inc-announces-buy-royalty-obligation-metabolix-inc/

2 https://www.fda.gov/drugs/postmarket-drug-safety-information-patients-and-providers/xyrem-sodium-oxybate-information

3 https://www.tepha.com/partnering/our-partners/

4 https://www.galateasurgical.com/surgical-scaffolds/
